# Precise Classification of Cervical Carcinomas Combined with Somatic Mutation Profiling Contributes to Predicting Disease Outcome

**DOI:** 10.1371/journal.pone.0133670

**Published:** 2015-07-21

**Authors:** Vivian M. Spaans, Marjolijn D. Trietsch, Alexander A. W. Peters, Michelle Osse, Natalja ter Haar, Gert J. Fleuren, Ekaterina S. Jordanova

**Affiliations:** 1 Department of Gynecology and Obstetrics, Leiden University Medical Center, Leiden, The Netherlands; 2 Department of Pathology, Leiden University Medical Center, Leiden, The Netherlands; 3 Department of Gynecology and Obstetrics, Center for Gynecologic Oncology, Amsterdam, The Netherlands; University of Quebec at Trois-Rivieres, CANADA

## Abstract

**Introduction:**

Squamous cell carcinoma (SCC), adenocarcinoma (AC), and adenosquamous carcinoma (ASC) are the most common histological subtypes of cervical cancer. Differences in the somatic mutation profiles of these subtypes have been suggested. We investigated the prevalence of somatic hot-spot mutations in three well-defined cohorts of SCC, AC, and ASC and determined the additional value of mutation profiling in predicting disease outcome relative to well-established prognostic parameters.

**Materials and Methods:**

Clinicopathological data were collected for 301 cervical tumors classified as SCC (*n*=166), AC (*n*=55), or ASC (*n*=80). Mass spectrometry was used to analyze 171 somatic hot-spot mutations in 13 relevant genes.

**Results:**

In 103 (34%) tumors, 123 mutations were detected (36% in SCC, 38% in AC, and 28% in ASC), mostly in *PIK3CA* (20%) and *KRAS* (7%). *PIK3CA* mutations occurred more frequently in SCC than AC (25% vs. 11%, *P*=0.025), whereas *KRAS* mutations occurred more frequently in AC than SCC (24% vs. 3%, *P*<0.001) and ASC (24% vs. 3%, *P*<0.001). A positive mutation status correlated with worse disease-free survival (HR 1.57, *P*=0.043). In multivariate analysis, tumor diameter, parametrial infiltration, and lymph node metastasis, but not the presence of a somatic mutation, were independent predictors of survival.

**Conclusion:**

Potentially targetable somatic mutations occurred in 34% of cervical tumors with different distributions among histological subtypes. Precise classification of cervical carcinomas in combination with mutation profiling is valuable for predicting disease outcome and may guide the development and selection of tumor-specific treatment approaches.

## Introduction

Cervical cancer is the third most common cancer and the fourth leading cause of cancer-associated death in women.[1] Based on histological features, the majority of invasive cervical carcinomas can be classified as squamous cell carcinoma (SCC), adenocarcinoma (AC), or adenosquamous carcinoma (ASC). SCC is the most common histological variant worldwide (~70%).[2] However, since the introduction of cytology-based screening programs, the incidence of SCC has declined in developed countries. In contrast, increasing incidence rates have been observed for AC and ASC, predominantly in younger women.[1,3–6] Differences in biological behavior, immune escape, tumor growth, metastasis, sensitivity to chemotherapy and radiotherapy, and prognosis have been observed between SCC, AC, and ASC.[4,7–9] In most studies concerning cervical cancer, the majority of cases are SCC, whereas AC and ASC are often combined into one A(S)C subgroup. To precisely classify AC, ASC, and SCC subtypes, additional mucus staining must be performed. However, the World Health Organization does not recommend routine mucus staining in clinical practice because it has not been shown to have any prognostic value.[2] Given the increasing incidence of AC and ASC, both absolute and relative to SCC, distinguishing between these histological subtypes is important and may contribute to the development of individualized tumor-specific treatment approaches.

Cervical cancer is caused by a persistent infection with oncogenic (high risk) type human papillomavirus (hrHPV), a DNA virus that infects the basal epithelium of the cervix.[10] HPV is a sexually transmitted virus with a lifetime risk of infection of approximately 80%. However, most infections are transient and efficiently cleared by the immune system. In about 10% persistence of infection occurs, which eventually can develop into premalignant cervical lesions and invasive cervical cancer.[11] The progression from persistent hrHPV infection into cervical cancer is determined by multiple factors.[10] During cervical carcinogenesis, various genetic and epigenetic events occur, such as loss of heterozygosity, tumor suppressor gene inactivation, and oncogene activation by point mutation or deletion.[12] A small number of studies have already reported genomic differences between the SCC and AC subtypes of cervical cancer, but there is no data on ASC, nor on the histological subtypes in the Dutch population.[13–15] Recently, we developed a high-throughput, mass spectrometry-based, somatic mutation profiling-panel specifically for gynecological malignancies. A total of 546 gynecological tumors, including 205 cervical carcinomas, were used to test and validate the panel. We showed that one or more somatic mutations occurred in 36% of cervical carcinomas, most of them in *PIK3CA* (24%), followed by *KRAS* (4%), *CTNNB1* (3%), and *PPP2R1A* (3%).[16]

We hypothesize that cervical SCC, AC and ASC might have different routes of malignant transformation. In the present study we aimed to determine and compare the somatic mutation profiles of cervical AC, ASC, and SCC. We retrospectively classified these histological subgroups based on morphology and specific mucus staining patterns and performed mutation analysis to determine the additive value of profiling somatic mutations in cervical tumors for predicting disease outcome. In total 301 cervical tumors were classified and analyzed for 171 somatic mutations in 13 genes. Here we present the results of this retrospective Dutch cohort analysis and discuss the possible impact of these results on the development and selection of future tumor-specific treatment approaches.

## Material and Methods

### Ethical statement

All human tissue samples used in this study were used according to the medical ethical guidelines described in the Code for Proper Secondary Use of Human Tissue, established by the Dutch Federation of Medical Sciences (http://www.federa.org; http://www.federa.org/sites/default/files/digital_version_first_part_code_of_conduct_in_uk_2011_12092012.pdf).[17] Patients receive information on, and can actively object against the secondary use of tissue that is sampled for diagnostic use. According to these guidelines, all human tissue samples were coded into anonymous data by the medical secretary of the department of Pathology. Because of this anonymization procedure, the Institutional Review Board of the Leiden University Medical Center confirmed that ethical approval was not required, and waived that individual patient’s consent was not required either.

### Patient samples

We included 320 patients with cervical carcinoma International Federation of Gynecology and Obstetrics (FIGO) stage IB-IIB [T1b-T2b N0 M0], who underwent radical hysterectomy with lymphadenectomy as primary treatment at the Leiden University Medical Center between January 1, 1985, and December 31, 2005, and from whom sufficient representative tumor tissue was available. Pathology reports were reviewed, and only tumors classified as SCC, AC, or ASC were selected. Clinical charts were reviewed retrospectively to collect data including age, FIGO stage, tumor diameter, stromal invasion depth, parametrial invasion, lymph-vascular space invasion (LVSI), tumor positivity of the resection margins, lymph nodes metastasis, HPV positivity and type, and adjuvant radiotherapy treatment. Follow-up data were collected until 60 months (5 years) after primary treatment, including disease recurrence and death by the tumor to determine disease-specific survival (DSS) from the primary surgery until tumor-related death or last follow-up (up to 60 months), and disease-free survival (DFS) from the primary surgery until disease recurrence or last follow-up (up to 60 months).

Formalin-fixed paraffin-embedded (FFPE) tissue blocks containing representative parts of the cervical tumor were retrieved from the archives of the department of Pathology, and histological sections were stained with hematoxylin and eosin for morphology. When no glandular components were seen, sections were stained with Periodic Acid Schiff Plus and Alcian Blue (PAS+/AB) to detect intra-cytoplasmic mucus. A well-experienced pathologist (GJF) reviewed the staining patterns to select subgroups and exclude unclear cases. SCC was defined as an invasive epithelial tumor composed of squamous cells at varying degrees of differentiation[2]; in this series all SCCs lacked glandular components, confirmed by negative PAS+/AB staining. AC was defined as an invasive epithelial tumor showing glandular differentiation (moderate to highly differentiated AC) or with positive PAS+/AB staining and lacking squamous elements (undifferentiated AC).[2] ASC was defined as an epithelial tumor comprising both SCC and glandular differentiation[2]; and glandular differentiation in undifferentiated cases was confirmed by PAS+/AB staining. Only usual type SCC, ASC, and AC were included; therefore, 17 tumors were excluded after primary selection (4 clear cell AC, 5 endometrioid type AC, 1 serous AC, 1 minimal deviation AC, 1 glassy cell AC, 1 small cell carcinoma, 2 secondary tumors, and 2 cases without remaining tumor tissue, only containing cervical intraepithelial neoplasia).

For DNA isolation, three to five 0.6 mm tissue cores were taken from a marked area of the FFPE tissue blocks containing ≥70% tumor. In the highly differentiated ACs, tumor cells were more diffusely positioned and micro-dissection was performed on 10 hematoxylin-stained 10-μm sections to achieve ≥70% tumor. DNA was isolated, followed by purification as described previously.[18–20] In two samples DNA isolation failed, and these cases were excluded from further analysis. A total of 301 samples remained for mutation genotyping.

### Somatic mutation genotyping

Somatic mutation genotyping was performed using the GynCarta 2.0 mutation panel (Sequenom, Hamburg, Germany) as described previously.[16] This panel analyzes mutations that are most commonly involved in gynecological malignancies, detecting 171 mutations in 13 genes: *BRAF*, *CDKN2A*, *CTNNB1*, *FBXW7*, *FGFR2*, *FGFR3*, *FOXL2*, *HRAS*, *KRAS*, *NRAS*, *PIK3CA*, *PPP2R1A*, and *PTEN*.

All 301 samples, plus 49 (16%) samples in duplicate and 11 (4%) samples in triplicate, were genotyped using the iPLEX technology system (Sequenom Inc., San Diego, USA) for matrix-assisted laser desorption/ionization time-of-flight mass spectrometry following the manufacturer’s protocol as described previously.[21] Non-template H_2_O samples (*N* = 14) and wild-type leukocyte DNA (*N =* 2) were included to obtain negative and wild-type spectra, respectively. Three investigators blinded to tumor identification analyzed the data using MassArray Typer Analyser software (TYPER 4.0.22, Sequenom, Hamburg, Germany) and MutationSurveyor (Softgenetics, State College, Pennsylvania, USA). The mutation spectra of 197 samples were published previously[16]; however, in that study we only described the mutation spectrum in the context of validating the mutation panel and no analysis concerning clinicopathological parameters or survival was performed.

### Statistical analysis

Statistical analyses were performed using IBM SPSS (Data Editor Version 20.0, Armonk, New York, USA). Baseline variables were compared with the chi-squared or Fishers’ exact test for categorical data. Normality was tested with the Shapiro-Wilk test; normally distributed continuous variables were compared using the independent Student *t-*test and skewed data were analyzed using the non-parametric Mann-Whitney U-test. Binary logistic regression was used to determine correlations between baseline characteristics and overall or gene-specific mutation status adjusting for clinicopathologic parameters for the total cohort and histological subgroups separately. Univariate Cox regression analysis determined correlations between overall mutation status or gene-specific mutation status and DSS. Multivariate Cox regression analysis assessed whether mutation status was independently associated with DSS. Univariate and multivariate analyses were repeated for DFS. Kaplan-Meier survival curves were generated using Graph Pad Prism (version 5.04). All *P*-values were two-sided and *P*<0.05 was considered significant, corresponding to 95% confidence intervals (CIs).

## Results

### Patients

A total of 301 cervical carcinoma patients were included in this study: 166 (55%) SCC, 55 (18%) AC, and 80 (27%) ASC. Patient and tumor characteristics are summarized in Table 1. Significant differences were observed between AC and ASC, and between AC and SCC. The AC cohort represented smaller, less invasive, less LVSI-positive tumors, which explains the lower rate of adjuvant radiotherapy in this cohort. Adjuvant radiotherapy was indicated in our clinic for patients with parametrial tumor infiltration, tumor-positive resection margins, lymph node metastasis, or two out of three of the following negative prognostic factors: positive LVSI, tumor size ≥ 40 mm, or tumor infiltration depth ≥ 15 mm.

**Table 1:** Clinicopathological parameters.

Characteristic	SCC		AC		ASC		*P-*value	*P*-value	*P-*value
	*n* = 166		*n* = 55		*n* = 80		SCC vs. AC	SCC vs. ASC	AC vs. ASC
Age (years), median (IQR)	44	(35–57)	42	(34–47)	43	(35–56)	**0.041**	0.588	0.162
FIGO stage I, *n* (%)	128	(78)	50	(91)	64	(80)	**0.034**	0.727	0.086
FIGO stage II	36	(22)	5	(9)	16	(20)			
Diameter (mm), median (IQR)	40	(30–50)	25	(17–35)	39	(25–55)	**0.000**	0.658	**0.000**
Infiltration (mm), median (IQR)	14	(10–19)	11	(5–15)	12	(9–18)	**0.001**	0.492	**0.011**
Parametria tumor-free, *n* (%)	137	(84)	52	(95)	69	(86)	**0.047**	0.654	0.120
Parametria infiltrated	26	(16)	3	(5)	11	(14)			
LVSI negative, *n* (%)	67	(42)	30	(71)	31	(40)	**0.001**	0.813	**0.001**
LVSI positive	93	(58)	12	(29)	46	(60)			
Lymph nodes negative, *n* (%)	113	(69)	44	(80)	58	(73)	0.114	0.565	0.319
Lymph nodes positive	51	(31)	11	(20)	22	(27)			
Margins tumor-free, *n* (%)	119	(73)	42	(76)	61	(77)	0.624	0.482	0.908
Margins infiltrated	44	(27)	13	(24)	18	(23)			
No radiotherapy, *n* (%)	60	(36)	33	(60)	34	(43)	**0.002**	0.337	**0.046**
Adjuvant radiotherapy	106	(64)	22	(40)	46	(58)			
hrHPV positive, *n* (%)	154	(93)	48	(87)	76	(95)	0.265	0.507	0.107
hrHPV negative	12	(7)	7	(13)	4	(5)			
HPV 16 positive, *n* (%)	102	(61)	24	(44)	39	(49)	**0.021**	0.059	0.558
HPV 18 positive	26	(16)	21	(38)	22	(28)	**0.000**	**0.028**	0.191

SCC, squamous cell carcinoma; AC, adenocarcinoma; ASC, adenosquamous carcinoma; FIGO, international federation of gynecology and obstetrics; LVSI, lymph vascular space invasion; (hr)HPV, (high risk) human papillomavirus; IQR, inter quartile range; *n* (%), number (percentage) of patients. Bold values are significant (*P*<0.05). Clinical data are missing for FIGO stage (*n* = 2), tumor diameter (*n* = 33), tumor infiltration depth (*n* = 13), parametrial infiltration (*n* = 3), LVSI (*n* = 22), lymph node metastasis (*n* = 2), and resection margins (*n* = 4). HPV was considered negative if no HPV DNA was detected or only low-risk type HPV (*n* = 1). In three tumors both HPV 16 and HPV 18 were detected.

### Mutation spectrum

**Fig 1:**
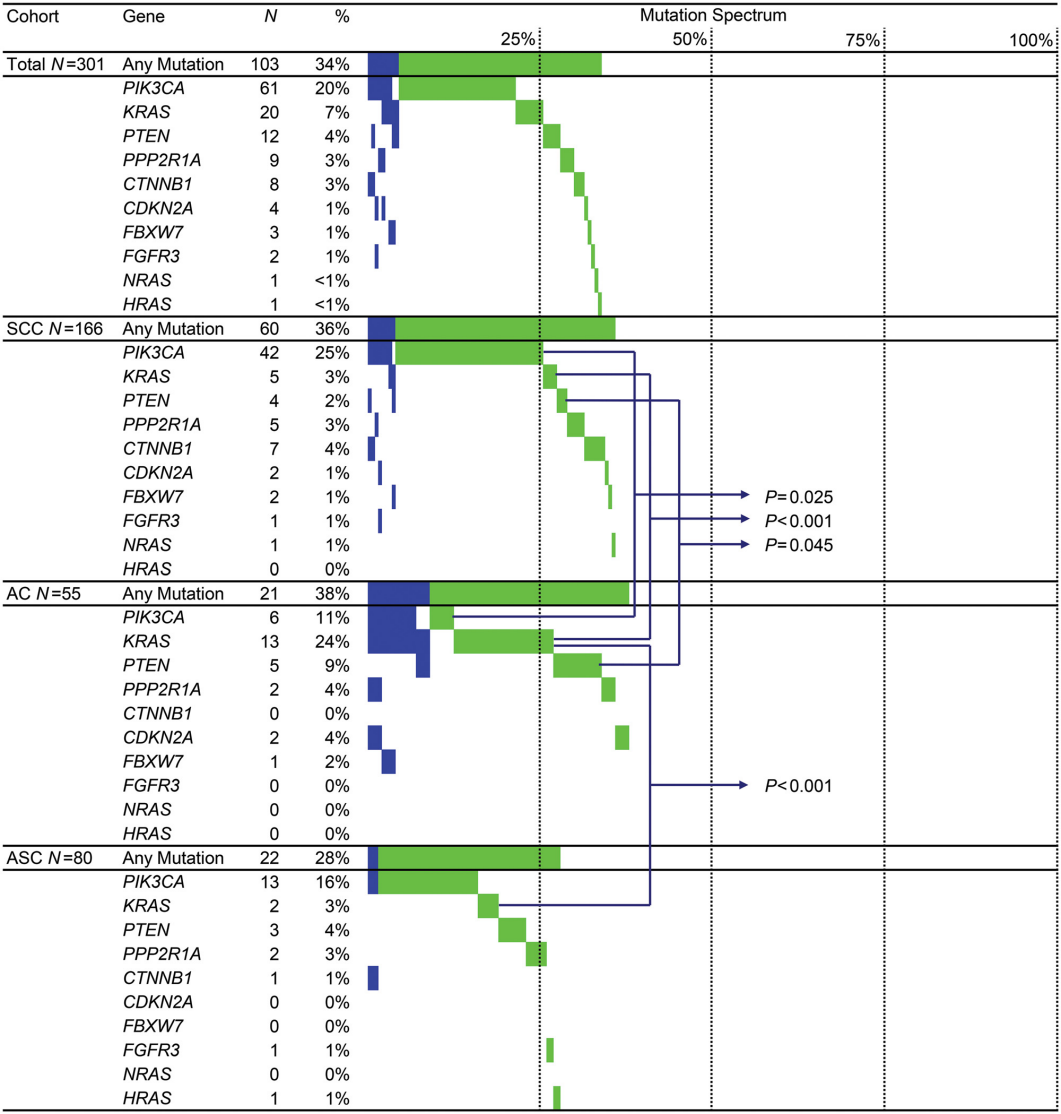
Mutation Spectrum. Mutation spectrum of 301 cervical cancers (top panel) and separate spectra for squamous cell carcinoma (SCC), adenocarcinoma (AC), and adenosquamous carcinoma (ASC). *N*, number of mutated samples; %, percentage mutated samples within the cohort. The mutation spectrum is visualized from left to right in percentages: blue bars, samples with ≥ 2 mutations; green bars, samples with a single mutation. Only significant *P-*values are shown between cohorts (all other values: see Table 2).

Mutation spectra of the histological subtypes are visualized in Fig 1, and mutation frequencies are summarized in Table 2. In 103 tumors (34%), 123 somatic mutations were detected. In 4% of the tumors more than one mutation was detected. No significant difference in somatic mutation prevalence was found between the histological subtypes (36% in SCC, 38% in AC, and 28% in ASC). However, a different mutation distribution was observed. *PIK3CA* mutations were detected more frequently in SCC than AC (*P* = 0.025). *KRAS* mutations were detected more frequently in AC than SCC (*P*<0.001) or ASC (*P<*0.001). *PTEN* mutations were detected in 4% of all tumors and most frequently in AC (9%). *CTNNB1* mutations were detected in 3% of all tumors and most frequently in SCC (4%).

**Table 2:** Mutation frequencies and distribution in 301 cervical carcinomas.

	All	SCC	AC	ASC	*P-*value	*P-*value	*P-*value
	*n* = 301	*n* = 166	*n* = 55	*n* = 80	SCC vs. AC	SCC vs. ASC	AC vs. ASC
**Any mutation, *n* (%)**	103 (34)	60 (36)	21 (38)	22 (28)	0.786	0.178	0.191
**>1 mutation, *n* (%)**	13 (4)	7 (4)	5 (9)	1 (1)	0.282	0.676	0.095
***PIK3CA*, *n* (%)**	61 (20)	42 (25)	6 (11)	13 (16)	**0.025**	0.110	0.381
p.E545K	40	29	3	8			
p.E542K	17	11	2	4			
p.H1047R	4	2	1	1			
p.R88Q	1	1	0	0			
***KRAS*, *n* (%)**	20 (7)	5 (3)	13 (24)	2 (3)	**0.000**	1.000	**0.000**
p.G12D	10	2	6	2			
p.G13S	4	0	4	0			
p.G12V	3	2	1	0			
p.G12C	1	1	0	0			
p.G12A	1	0	1	0			
p.G12S	1	0	1	0			
***PTEN*, *n* (%)**	12 (4)	4 (2)	5 (9)	3 (4)	**0.045**	0.685	0.270
p.R130G	3	1	2	0			
p.Q214*	2	1	0	1			
p.R234W	2	1	0	1			
p.R130*	1	1	0	0			
p.R130fs*4	1	0	1	0			
p.R173H	1	0	1	0			
p.K267fs*31	1	0	1	0			
p.K267fs*9	1	0	0	1			
***PPP2R1A*, *n (%)***	9 (3)	5 (3)	2 (4)	2 (3)	1.000	1.000	1.000
p.R258H	6	3	1	2			
p.P179R	3	2	1	0			
p.R183W	1	1	0	0			
***CTNNB1*, *n* (%)**	8 (3)	7 (4)	0 (0)	1 (1)	0.197	0.443	1.000
p.G34R	3	2	0	1			
p.D32N	1	1	0	0			
p.S37F	1	1	0	0			
p.S45F	1	1	0	0			
p.T41A	1	1	0	0			
p.T41I	1	1	0	0			
***CDKN2A*, *n* (%)**	4 (1)	2 (1)	2 (4)	0 (0)	0.259	1.000	0.164
p.R58*	2	1	1	0			
p.P114L	1	1	0	0			
p.W110*	1	0	1	0			
***FBXW7*, *n* (%)**	3 (1)	2 (1)	1 (2)	0 (0)	1.000	1.000	0.407
p.R465H	2	2	0	0			
p.R465C	1	0	1	0			
***FGFR3*, *n* (%)**	2 (1)	1 (1)	0 (0)	1 (1)	1.000	0.546	1.000
p.S249C	2	1	0	1			
***NRAS*, *n* (%)**	1 (<1)	1 (1)	0 (0)	0 (0)	N.A.	N.A.	N.A.
p.Q61R	1	1	0	0			
***HRAS*, *n* (%)**	1 (<1)	0 (0)	0 (0)	1 (1)	N.A.	N.A.	N.A.
p.G12D	1	0	0	1			
***FGFR2*, *n* (%)**	0 (0)	0 (0)	0 (0)	0 (0)	N.A.	N.A.	N.A.
***BRAF*, *n* (%)**	0 (0)	0 (0)	0 (0)	0 (0)	N.A.	N.A.	N.A.
***FOXL2*, *n* (%)**	0 (0)	0 (0)	0 (0)	0 (0)	N.A.	N.A.	N.A.

SCC, squamous cell carcinoma; AC, adenocarcinoma; ASC, adenosquamous carcinoma. N.A. statistical test not applicable. Frequencies are given as number *(n*) of samples with the mutation—one SCC sample contains two *PPP2R1A* mutations (p.P179R and p.R183W) and one SCC sample contains two *PIK3CA* mutations (p.E542K and p.E545K). *P-*values were determined by chi-square test or Fisher's exact test; bold *P-*values are considered significant (< 0.05).

### Correlations between mutation status and clinicopathological characteristics

Binary logistic regression analysis assessed whether the overall somatic mutation status and gene-specific mutation status correlated with clinicopathological parameters for the total patient cohort or within the SCC, AC, and ASC subgroups. Overall, patients with a positive somatic mutation status were significantly older at disease onset (mean age 49 vs. 45 years; *P* = 0.004). This feature was explained by the strong association between older age and *PIK3CA* mutation in SCC patients (mean age 53 vs. 45 years; *P =* 0.003). In addition, having a *PIK3CA* mutation was associated with FIGO stage II in the SCC subtype (42% vs. 15%; *P =* 0.001). In the AC subtype, *PIK3CA* mutation correlated with tumor diameter (mean diameter in *PIK3CA-*mutated samples 40 mm vs. 26 mm in non-*PIK3CA* mutated samples; *P* = 0.011). In the ASC subtype, having any mutation also correlated with older age (mean age 49 vs. 45 years; *P =* 0.048), parametrial infiltration (23% vs. 10%; *P =* 0.024), and inversely correlated with LVSI (36% vs. 69%; *P =* 0.002).

For the whole cohort and within histological subgroups, a positive mutation status was not significantly correlated with hrHPV positivity/negativity or type. Gene specifically hrHPV negativity correlated with a *PTEN* mutation (5/23 *PTEN* mutations in hrHPV negative tumors vs. 7/278 *PTEN* mutations in hrHPV positive tumors, *P<*0.001). This association between *PTEN* mutation and hrHPV negativity was also detected in SCC patients separately (3/12 vs. 1/154, *P<*0.001), but not found in AC and ASC patients. Within the SCC cohort also *CDKN2A* (1/12 vs. 1/154, *P =* 0.019), *FBXW7* (1/12 vs. 1/154, *P =* 0.019), *FGFR3* (1/12 vs. 0/154, *P<*0.001) mutations significantly correlated with hrHPV negativity. In 5/12 hrHPV negative SCC patients 11 somatic hot-spot mutations were detected. In one tumor a single *PIK3CA* E545K mutation was detected, in one tumor a single *PTEN* R234W mutation was detected, and in three of these tumors three different mutations were detected. One with a combination of *PIK3CA* p.R88Q, *PTEN* p.Q214*, and *CTNNB1* p.S45F, one with a combination of *PIK3CA* p.E545K, *CDKN2A* p.R58*, and *FGFR3* p.S249C, and one with the combination of *KRAS* p.G12V, *PTEN* p.R130G, and *FBXW7* p.R465H. Within the AC and ASC patient cohorts no correlations were detected between mutation status and hrHPV. Also, no correlations were detected per hot-spot mutation or hrHPV type specifically; probably due to small numbers. In Table 3 all hrHPV types detected in this series per hot-spot mutation are shown (Table 3).

**Table 3:** High risk Human Papillomavirus types per hot-spot mutation and per histological subtype.

Gene	Mutation	SCC	AC	ASC
***PIK3CA*, *n = 61* (20%)**	p.E545K, *n =* 40	negative (2)	16	16 (4)
		16 (15)	18 (2)	18
		18 (4)		31
		45		33
		51		16+68
		52		
		73		
		16+31		
		16+68+73		
		18+45		
		51+52		
	p.E542K, *n =* 17	16 (8)	16 (2)	16 (3)
		18		31
		31		
		52		
	p.H1047R, *n =* 4	31	18	51
		33		
	p.R88Q, *n =* 1	negative		
***KRAS*, *n = 20* (7%)**	p.G12D, *n =* 10	16	negative (2)	18 (2)
		52	16 (3)	
			18	
	p.G13S, *n =* 4		16 (3)	
			18	
	p.G12V, *n =* 3	negative	18	
		16		
	p.G12C, *n =* 1	16		
	p.G12A, *n* = 1		16	
	p.G12S, *n* = 1		18	
***PTEN*, *n = 12* (4%)**	p.R130G, *n* = 3	negative	negative (2)	
	p.Q214*, *n* = 2	negative		16
	p.R234W, *n* = 2	negative		16
	p.R130*, *n* = 1	16		
	p.R130fs*4, *n* = 1		18	
	p.R173H, *n* = 1		16	
	p.K267fs*31, *n* = 1		16+18	
	p.K267fs*9, *n* = 1			16
***PPP2R1A*, *n = 9 (3%)***	p.R258H, *n* = 6	16	16	16
		16+52		18
		31		
	p.P179R, *n* = 3	16	18	
		33		
	p.R183W, *n* = 1	16		
***CTNNB1*, *n = 8* (3%)**	p.G34R, *n* = 3	16		16
		45		
	p.D32N, *n* = 1	16		
	p.S37F, *n* = 1	16		
	p.S45F, *n* = 1	negative		
	p.T41A, *n* = 1	33		
	p.T41I, *n* = 1	16		
***CDKN2A*, *n = 4* (1%)**	p.R58*, *n* = 2	negative	45	
	p.P114L, *n* = 1	16		
	p.W110*, *n* = 1		16	
***FBXW7*, *n = 3* (1%)**	p.R465H, *n* = 2	negative		
		16		
	p.R465C, *n* = 1		18	
***FGFR3*, *n = 2* (1%)**	p.S249C, *n* = 2	negative		16
***HRAS*, *n = 1* (<1%)**	p.G12D, *n* = 1			16+33
***NRAS*, *n = 1 (<1*%)**	p.Q61R, *n* = 1	16		

SCC, squamous cell carcinoma; AC, adenocarcinoma; ASC, adenosquamous carcinoma. When more than one tumor with the same mutation and same HPV type was detected, the number is given between brackets. Seven tumors were double positive, one tumor was triple positive, shown with"+".

### Correlations between mutation status and survival

Within 5 years of primary surgery, 81 (27%) patients suffered from recurrent disease (mean DFS 48 months) and 53 (18%) patients died from cervical cancer (mean DSS 53 months).

**Fig 2:**
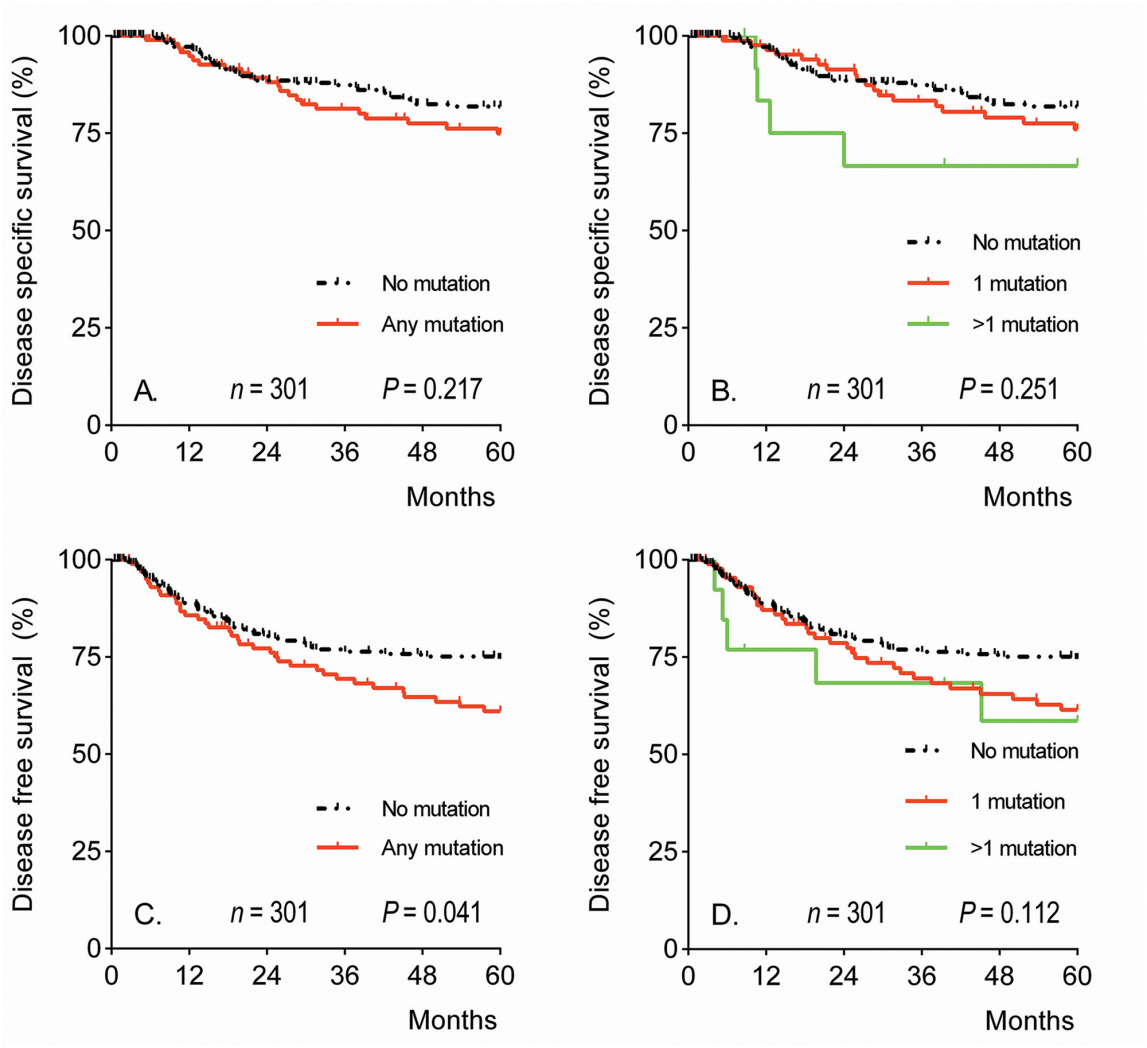
Five-year survival curves for all cervical cancer patients by mutational status. Five-year disease-specific (A and B) and disease-free (C and D) Kaplan-Meier survival curves based on overall mutation status (A and C) and multiple mutations (B and D). *P*-values were calculated by the Log Rank-test.

Univariate survival analyses were performed to determine the correlations between mutational status, histology, and other clinicopathological characteristics and DSS or DFS (Table 4). No difference in DSS was found for patients with tumors with any somatic mutation compared to patients without any somatic mutation (Hazard Radio (HR) 1.41, 95% CI 0.82–2.43); however, for DFS this difference was significant (HR 1.57, 95% CI 1.01–2.44; Fig 2). Subsequently, multivariate Cox survival analyses were performed for DSS and DFS and showed that tumor diameter and lymph nodes metastasis were independent predictors of DSS and tumor diameter, lymph nodes metastasis and parametrial infiltration were independent predictors of DFS (Table 4). A positive somatic mutation status was not an independent predictor of DSS or DFS.

**Table 4:** Univariate and multivariate Cox regression analysis for disease-specific and disease-free survival.

Variable	Disease-specific survival						Disease-free survival					
	Univariate analysis			Multivariate analysis			Univariate analysis			Multivariate analysis		
	HR	95% CI	*P*-value	HR	95% CI	*P-*value	HR	95% CI	*P*-value	HR	95% CI	*P-*value
Mutations (≥1)	1.41	0.82–2.43	0.219	1.06	0.57–1.98	0.845	1.57	1.01–2.44	**0.043**	1.24	0.76–2.04	0.390
SCC	(ref)	—	0.102				(ref)	—	0.222			
AC	0.41	0.16–1.04	0.061				0.58	0.30–1.12	0.102			
ASC	1.19	0.66–2.15	0.562				1.04	0.63–1.71	0.876			
Age at disease onset (years)	1.00	0.98–1.02	0.780				1.00	0.99–1.02	0.762			
Tumor diameter (mm)	1.03	1.02–1.04	**0.000**	1.03	1.02–1.05	**0.000**	1.03	1.02–1.04	**0.000**	1.03	1.02–1.04	**0.000**
Infiltration depth (mm)	1.04	1.02–1.07	**0.001**	0.99	0.96–1.03	0.666	1.04	1.02–1.06	**0.000**	1.01	0.98–1.04	0.498
Parametrial infiltration	4.83	2.69–8.66	**0.000**	1.96	0.95–4.04	0.069	3.73	2.26–6.16	**0.000**	2.12	1.15–3.88	**0.015**
Lymph node metastasis	4.19	2.43–7.24	**0.000**	3.34	1.74–6.40	**0.000**	3.14	2.02–4.89	**0.000**	2.43	1.44–4.09	**0.001**

SCC, squamous cell carcinoma; AC, adenocarcinoma; ASC, adenosquamous carcinoma; HR, hazard ratio; 95% CI, 95% confidence interval of hazard ratio. Calculated by univariate and multivariate Cox regression analysis for survival, *P-*values in bold are considered significant (<0.05).

**Fig 3:**
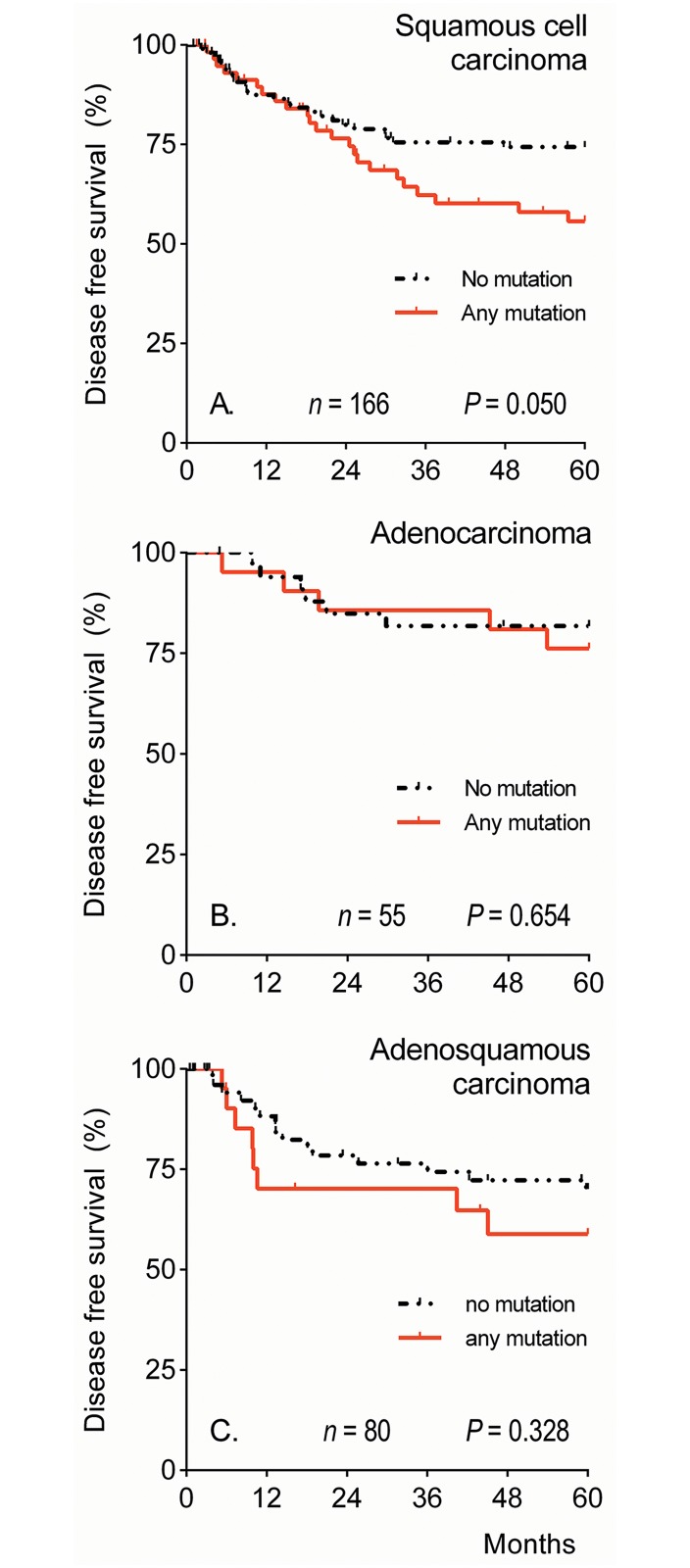
Five-year disease-free survival by mutational status per histological subtype. Five-year disease-free Kaplan-Meier survival curves for cervical squamous cell carcinoma (A), cervical adenocarcinoma (B), and cervical adenosquamous carcinoma (C) patients based on mutational status. *P*-values were calculated by the Log Rank-test.

Univariate and multivariate DSS and DFS analyses were repeated for SCC, AC or ASC separately. The Kaplan Meier curves for the DFS are shown in Fig 3. For SCC, but not AC or ASC, a trend was seen for disease recurrence for patients with a positive mutation status (DFS HR 1.76, 95% CI 0.99–3.12; Fig 3A). For SCC, in multivariate analysis tumor diameter and parametrial infiltration were independent predictors of DSS (HR 1.03, 95% CI 1.01–1.05, HR 2.89, 95% CI 1.11–7.55, respectively) and DFS (HR 1.02, 95% CI 1.01–1.04, HR 3.08, 95% CI 1.40–6.76, respectively). For AC, in multivariate analysis tumor diameter and lymph node metastasis were independent predictors of DSS (HR 1.10, 95% CI 1.01–1.20, HR 34.21, 95% CI 2.63–445.7, respectively) and DFS (HR 1.07, 95% CI 1.02–1.12, HR 6.08, 95% CI 1.70–21.81, respectively). For ASC, in multivariate analysis only lymph node metastasis was an independent predictor of DSS (HR 5.24, 95% CI 1.73–15.93) and lymph node metastasis as well as tumor diameter were independent predictors of DFS (HR 2.90, 95% CI 1.13–7.41, HR 1.02, 95% CI 1.00–1.04, respectively).

**Fig 4:**
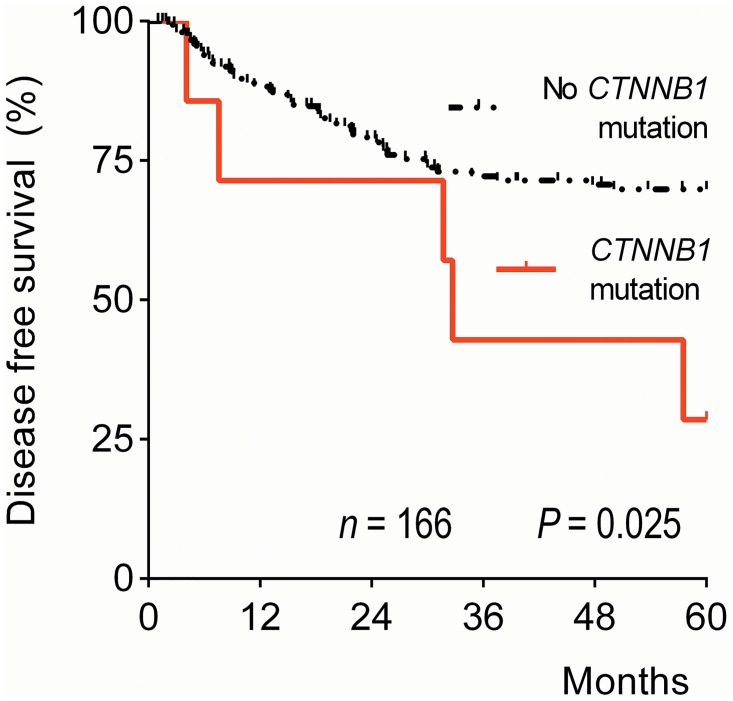
Five-year disease-free survival curve by *CTNNB1* gene mutation status for cervical squamous cell carcinoma patients. The *P-*value was calculated by the Log Rank-test.

We determined the correlation between DSS/DFS and gene-specific mutational status for all genes in which mutations were detected for the whole cohort and within histological subgroups. Univariate analysis revealed an increased risk of disease recurrence, but not DSS, in SCC patients with a *CTNNB1* mutation (DSS HR 2.39, 95% CI 0.73–7.87; DFS HR 2.76, 95% CI 1.09–6.98; Fig 4). In ASC, an *HRAS* mutation was associated with worse DSS (HR 34.67, 95% CI 3.14–382.26) and DFS (HR 13.62, 95% CI 1.59–116.62). However, as only one *HRAS* mutation was detected in the whole series, giving a broad confidence interval, this result should be questioned. Multivariate analysis again showed that tumor diameter, parametrial infiltration, and lymph node metastasis, but none of the distinct genes, were independent predictors of survival and disease recurrence (data not shown).

## Discussion

All cancer genomes carry genetic aberrations,[22] and studies concerning various carcinomas have shown that somatic mutation profiling can be useful for discriminating between tumor subtypes, predicting prognosis, and identifying new drug targets and tumor-specific treatment options.[23–27]

Cervical cancer is caused by a persistent infection with hrHPV, but multiple factors are involved in its development, including somatic mutations. In the present study, we demonstrated that somatic hotspot mutations occur in 34% of cervical tumors, with different distributions among histological subtypes and correlating with several tumor characteristics. These results imply that, in cervical cancer, somatic mutations play an important role in oncogenesis and mutation profiling of cervical carcinomas may contribute to the design and selection of tumor targeting therapies.

Increasing incidence of cervical AC and ASC relative to SCC stresses the need for investigation of the oncogenic differences between these histological subtypes.[3–5,9] In the present study, we classified 301 cervical tumors by additional PAS+/AB staining, leading to a relatively higher percentage of A(S)C (45%) compared to established percentages (SCC 70%, A(S)C 10–25%).[2] We investigated the frequencies and distribution of 171 somatic mutations in 13 genes. Differences in oncogenic mutations between cervical SCC and AC were described previously in patients from the United States, Norway, and Mexico.[14,15] Previously, we reported the mutation spectrum of 205 Dutch cervical tumors,[16] but that study was performed to describe the mutation spectrum and validate a test panel. In contrast, the present study comprises more tumor samples and was performed to analyze correlations with clinicopathological parameters and survival. To the best of our knowledge, mutations have not previously been described separately in a large ASC cohort. The mutation panel used in our study was designed specifically for gynecological malignancies[16] based on previously reported mutations in gynecological tumors, giving a more precise overview of mutations compared to studies using generic cancer gene panels.[14,28] However, because of the mass spectrometric method we used, only “hot-spot” mutations were included in the panel, with which high coverage could be achieved.[16] Mutations in *TP53* were not included in this study, and therefore 34% of somatic mutations in this cohort is likely an underestimation. *TP53* is mutated in approximately 5% of all cervical tumors according to the COSMIC online database.[29] Different patterns of *TP53* mutations have been described in SCC and AC in different regions of the world, with the highest frequency of *TP53* mutations occurring in AC patients in Asia.[13] However, *TP53* mutations are widely scattered throughout the gene and, therefore, not suitable for analysis by the approach used here.

The detected *PIK3CA* mutations were predominantly p.E545K and p.E542K mutations, similar to previous results,[30] and only four p.H1047R mutations were detected. *PIK3CA* mutations lead to deregulation of the phosphatidylinositol 3-kinase-Akt signaling pathway, which comprises cell proliferation, transformation, and cell survival, stimulating oncogenesis. Aberrations in this pathway are described in many cancers, including cervical cancer, and this has led to the development of PI3K-inhibitors and Akt-inhibitors as potential cancer therapies, with some already having reached clinical trials.[31–34] Mammalian target of rapamycin (mTOR) is a key protein downstream the PI3K-Akt pathway and mTOR-targeting agents (everolimus, temsirolimus) are currently used for several cancers in clinical practice.[35–38] The role that PI3K-Akt pathway targeting can play in cervical cancer therapy remains to be investigated further.[39]

*PIK3CA* mutation rates are very heterogeneous in different studies (20–37%).[14,39–42] Controversial results as to the mutation frequencies in different histological types have been reported possibly due to the different techniques used, or due to differences in population genetics. In our study, *PIK3CA* was the predominant gene mutated in SCC (25%), and we show that *PIK3CA* was also frequently mutated in ASC and AC (16% and 11%, respectively). Furthermore, the presence of a *PIK3CA* mutation specifically correlated with advanced age at disease onset in SCC in this Dutch cervical cancer cohort. Cui et al. and McIntyre et al. previously reported this feature in Swedish and Canadian cervical cancer cases, respectively,[40,43] and it has also been described in other cancers.[44,45]

Studies in lung and colorectal cancer have demonstrated that *KRAS* mutations are associated with reduced effects of PI3K/Akt/mTOR therapies.[46,47] A *KRAS* mutation was detected in 24% of cervical AC patients compared to only 3% of SCC and 3% of ASC patients. A similar difference between cervical AC and SCC was reported by others.[14] Importantly, we classified the tumor subtypes more precisely than previous studies.

*KRAS* mutations are detected and investigated primarily in colorectal cancer, pancreatic cancer, and lung cancer. Targeted therapies blocking *KRAS* itself have not yet been developed, but recent studies targeting RAS signaling are promising.[48,49] Furthermore, colorectal cancer patients with a *KRAS* mutation respond less to EGFR-targeting drugs.[26] In non-small cell lung cancer, this association has not been demonstrated, but *KRAS* mutations are associated with worse prognosis.[50] The role of *KRAS* mutations in cervical AC remains to be investigated.

In the present study, neither *KRAS* nor *PIK3CA* mutations were associated with survival, although a clear trend was seen for reduced survival in patients carrying a *PIK3CA* mutation, especially with the SCC subtype. However, *CTNNB1* mutations in SCC patients were associated with disease recurrence in this cohort. *CTNNB1* encodes the beta-catenin protein, which is responsible for cell-cell adhesion and intracellular signaling downstream in the Wnt pathway. Mutated *CTNNB1* acts as an oncogene, stimulating cell proliferation and inhibiting apoptosis. *CTNNB1* is studied most intensively in colorectal and hepatocellular cancer but has also been described in other cancer types, such as ovarian and endometrial cancer.[51,52] In cervical cancer, *CTNNB1* mutations have been detected in 3 out of 15 SCC cell lines,[53,54] but the mutations have never been described in association with survival. Although only 4% of all SCCs had *CTNNB1* mutations, the significant association with disease recurrence warrants further investigation. The Wnt/beta-catenin signaling pathway is a popular target for pharmacological research and has led to the development of several Wnt inhibitors; yet, so far no therapies are currently used in clinical practice.[52,55]

In this study, we have identified the most frequent, potentially targetable, somatic mutations among the three most common histological subtypes of cervical cancer. Differences in the somatic mutation profiles of these subtypes suggest different routes of malignant transformation. In clinical practice, cervical AC, ASC, and SCC subtypes are classified based only on their histological features, but additional mucus staining is not recommended because the tumor’s subtype has no consequences for the treatment dogma. In the present study, we have carefully classified the subtypes using additional mucus staining, and we show clear mutation spectrum differences between the histological subtypes resulting in a landscape of potentially targetable mutations in cervical cancer. These results may contribute to the future development and selection of tumor-specific treatment approaches. Undoubtedly, in the near future additional mutations will be detected by the ongoing international exome- and full-genome sequencing consortia that could be added to panel of histology-specific genes crucial in cervical cancer development and prognosis.
